# ZnO–Ti_3_C_2_ MXene Electron Transport Layer for High External Quantum Efficiency Perovskite Nanocrystal Light‐Emitting Diodes

**DOI:** 10.1002/advs.202001562

**Published:** 2020-08-16

**Authors:** Po Lu, Jinlei Wu, Xinyu Shen, Xupeng Gao, Zhifeng Shi, Min Lu, William W. Yu, Yu Zhang

**Affiliations:** ^1^ State Key Laboratory of Integrated Optoelectronics and College of Electronic Science and Engineering Jilin University Changchun 130012 China; ^2^ Key Laboratory of Materials Physics of Ministry of Education School of Physics and Microelectronics Zhengzhou University Zhengzhou 450052 China; ^3^ Department of Chemistry and Physics Louisiana State University Shreveport LA 71115 USA

**Keywords:** light‐emitting diodes, MXenes, perovskite nanocrystals, Ti_3_C_2_

## Abstract

2D transition metal carbides, nitrides, and carbonitrides called MXenes show outstanding performance in many applications due to their superior physical and chemical properties. Herein, a ZnO–MXene mixture with different contents of Ti_3_C_2_ is applied as electron transport layers (ETLs) and the influence of the Ti_3_C_2_ MXene in all‐inorganic metal halide perovskite nanocrystal light‐emitting diodes (perovskite NC LEDs) is explored. The addition of Ti_3_C_2_ makes more balanced charge carrier transport in LEDs by changing the energy level structure and electron mobility of ETL. Moreover, lower surface roughness is obtained for the ETL, thus guaranteeing uniform distribution of the perovskite NCs layer and further reducing leakage current. As a result, a 17.4% external quantum efficiency (EQE) with low efficiency roll‐off is achieved with 10% Ti_3_C_2_, which is a 22.5% improvement compared to LEDs without Ti_3_C_2_.

## Graphical abstract

Adding an appropriate amount of Ti_3_C_2_ into the ZnO electron transport layer (ETL) to change the energy level structure and electron mobility of the ETL and further make the carrier transport balanced is explored. The ZnO/TiC ETL‐based red emitting perovskite nanocrystals light‐emitting diodes exhibit an external quantum efficiency of 17.4%.

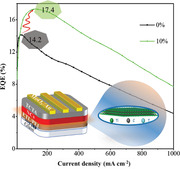

All‐inorganic metal halide perovskite nanocrystals (NCs) have very recently emerged as interesting materials for optoelectronic applications because of their superior properties (e.g., long charge carrier diffusion length, high photoluminescence quantum yields (PL QYs), tunable bandgaps over the entire visible spectral range, and facile synthesis).^[^ ^1^ ^]^ In addition, they have wider color gamut and higher color purity than II–VI NCs and organic fluorophores.^[^ ^2^ ^]^ Based on the above advantages, their application in light‐emitting diodes (LEDs) is also progressing rapidly. So far, the external quantum efficiency (EQE) of CsPbX_3_ NC LEDs has exceeded 20%,^[^ ^3^ ^]^ which makes them very competitive in the application of next‐generation display technology.

LED's EQE can be expressed as^[^ ^4^ ^]^ EQE = *η*_out_ *χγη*_PL_, where *η*_out_ is the optical outcoupling coefficient, *γ* is the fraction of excitons that can radiatively decay, *η*_PL_ is the PL QY of emitting layer (EML), *χ* is the balance of charge injection. Based on the above formula, when only the internal factors of the device are considered, the factors affecting the EQE of LEDs can be mainly divided into two aspects: i) the influence of the light‐emitting layer materials; ii) the design of the device structure. For the former, the strategy of ligand engineering or element doping is basically used to increase the radiative recombination probability of electrons and holes, thereby improving the PL QY.^[^ ^5^ ^]^ For the latter, a lot of research works have been focused on device structure optimization to guarantee balanced charge injection, which needs appropriate energy band alignment and equivalent carrier mobility of charge transport layer (CTL) materials. For instance, Kim et al. utilized a perfluorinated polymeric acid (tetrafluoroethylene‐perfluoro‐3,6‐dioxa‐4‐methyl‐7‐octene‐sulfonic acid copolymer (PFI)) to modify PEDOT:PSS, which forms a self‐assembled gradient work function between PEDOT:PSS and perovskite, thereby reducing the hole injection barrier between them. Similarly to PFI, ethanolamine (EA)‐modified TiO_2_, polyethyleneimine (PEI)‐modified ZnO, polyethylenimine ethoxylated (PEIE)‐modified ZnO, and PFI modified poly‐TPD have been applied in perovskite LEDs (PeLEDs) to achieve a balanced charge injection and further improving the device performance.^[^ ^6^ ^]^ In addition, Wu et al. and Zhang et al. used Mg‐doped ZnO and Li‐doped TiO_2_ as the electron transport layers (ETLs) to reduce the electron injection barrier and the carrier mobility, making the electron and hole injection more balanced. Therefore, the device performances were significantly improved.^[^ ^7^ ^]^ Sim et al. prepared amorphous Zn–Si–O ETL by magnetron sputtering at room temperature, which had sufficiently shallow electron affinity (≈3.2 eV)  to confine excitons and sufficiently high electron mobility (≈0.8 cm^2^ V^−1^ s^−1^) to transport electrons. Consequently, the very low operating voltage and high power efficiency were achieved for CsPbX (X=Br_3_ or BrI_2_) perovskite LEDs.^[^ ^8^ ^]^

Recently, 2D transition metal carbides, nitrides, and carbonitrides called MXenes with a formula of M*_n_*_+1_X*_n_* T*_x_* (*n* = 1, 2, 3; M is an early transition metal, X is carbon and/or nitrogen atom and T*_x_* represents the surface‐terminating functional groups) show outstanding performance in many applications such as supercapacitors, catalysts, conducting thin films, sensors, and antennas due to their superior physical and chemical properties.^[^ ^9^ ^]^ In addition, MXenes offer relatively high conductivity, high light transmittance in the visible range, and the possibility to tailor their electronic structure such as work function (WF) or bandgap.^[^ ^10^ ^]^ All of these make MXenes possible to be the transport material in optoelectronic applications. Ti_3_C_2_T*_x_* MXenes have been incorporated into the perovskite absorber or into the ETL, modifying the chemical and/or physical properties of the interface and providing superior charge transfer paths, therefore, resulting in an enhanced power conversion efficiency in perovskite solar cells.^[^ ^11^ ^]^ Considering the interoperability of them, it is also possible to apply the MXenes to LEDs.

Herein, sheet‐like Ti_3_C_2_ was incorporated into the ZnO ETL for CsPb_0.64_Zn_0.36_I_3_ NC LEDs. ETL films of ZnO with different Ti_3_C_2_ contents were prepared to explore its impact on device performance. It can be seen that the addition of Ti_3_C_2_ significantly changes the energy level structure of the ETL and affects the carrier transport balance. The highest EQE up to 17.4% was achieved for 10% Ti_3_C_2_ based LEDs, which is 22.5% improvement compared with the ZnO‐based device (14.2% EQE). In addition, the efficiency roll‐off in high current density was also effectively improved due to the more balanced carrier recombination. To our best knowledge, this is the first work to study the influence of Ti_3_C_2_ MXene on device performances of perovskite LEDs.

The fabrication route of Ti_3_C_2_ MXene is illustrated in **Figure** **1**a. Ti_3_C_2_ MXene was obtained through etching bulk Ti_3_AlC_2_ ceramics by a mixed solution of LiF salt and HCl acid. The accordion‐like Ti_3_C_2_ was further exfoliated into Ti_3_C_2_ sheets via ultrasound (details in the Supporting Information). Figure 1b,c shows the electron microscope (TEM) and high‐resolution TEM (HR‐TEM) images of the as‐synthesized Ti_3_C_2_ MXene, respectively. The sheet‐like Ti_3_C_2_ MXene has a rather flat and smooth surface with an average edge length of several hundred nanometers (Figure 1b). The edges (approximately two layers) are spontaneously folded due to their high flexibility. The XRD of Ti_3_AlC_2_ (JCPDS 52‐0875) and Ti_3_C_2_ MXene are shown in Figure 1d. The strongest diffraction peak around 38.9°, corresponding to the (104) plane of the original material Ti_3_AlC_2_, disappeared after etching out the Al layer. Meanwhile, the (002) plane at 9.54° and (004) plane at 19.1° of Ti_3_AlC_2_ were broadened and shifted to smaller angles, indicating the formation of Ti_3_C_2_ sheets.^[^ ^11b^ ^]^

**Figure 1:**
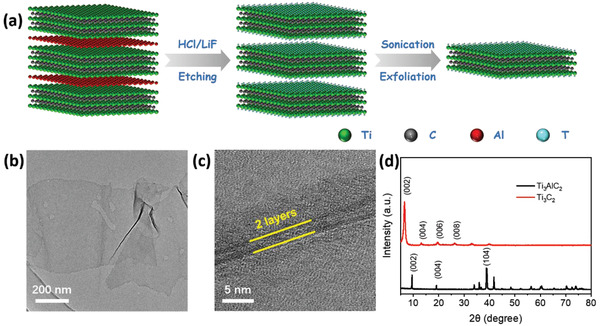
a) Schematic diagram for the formation of sheet‐like Ti_3_C_2_. b) TEM and c) HR‐TEM images of Ti_3_C_2_. d) XRD patterns of Ti_3_AlC_2_ and Ti_3_C_2_.

For perovskite EML, the synthesis of CsPb_0.64_Zn_0.36_I_3_ perovskite NCs was based on our previous work using a 0.5 feed ratio of ZnI_2_ to PbI_2_ (details in the Supporting Information). Figure S2a (Supporting Information) shows the UV−vis absorption and photoluminescence (PL) spectra of the as‐synthesized CsPb_0.64_Zn_0.36_I_3_ perovskite NCs whose first excitonic absorption peak and PL peak are 675 nm  and 688 nm,  respectively. The corresponding XRD patterns and TEM images are shown in Figure S2b,c (Supporting Information), proving the perovskite NCs with a cubic phase.

The ZnO–Ti_3_C_2_ (designated as ZTC) mixture are made up of the different amounts of Ti_3_C_2_ MXene dispersed into the ZnO NC solution. As shown in Figure S2 (Supporting Information), they formed a uniform dispersion and the color of the solution gradually darkened as the amount of Ti_3_C_2_ increased. The XRD patterns of different samples are shown in Figure S3 (Supporting Information). Figure S4a (Supporting Information) shows the TEM image of the ZTC. It can be seen that ZnO is attached to or scattered around the surface of Ti_3_C_2_. Figure S4b (Supporting Information) is the diffraction pattern without the Ti_3_C_2_ area and the diffraction rings of ZnO can be seen. Figure S4c (Supporting Information) is the diffraction pattern of the mixed region. Compared with Figure S4b (Supporting Information), there are two more obvious diffraction rings, corresponding to the (101) and (110) planes of Ti_3_C_2_,^[^ ^12^ ^]^ indicating that there is indeed Ti_3_C_2_ in the mixed region. SEM images of unheated and heated ZTC are shown in Figure S4d,e (Supporting Information), respectively. Figure S4d (Supporting Information) shows that a small amount of sheet‐like Ti_3_C_2_ is mixed on the ZnO film, and Figure S4e (Supporting Information) shows that the annealed film has uniform grains. In the process of device preparation, ZTC mixture were spin‐coated onto an ITO substrate as ETL and a light output medium. In order to guarantee a high optical coupling output of the device, the light transmittance of the spin‐coated ZTC mixture film cannot be greatly reduced. Subsequently, the transmittances of pristine ZnO film and ZTC film with different amounts of Ti_3_C_2_ were characterized and presented in Figure S5 (Supporting Information). There is no significant change in transmittance between ZTC and pristine ZnO film over the entire visible range, demonstrating that the introduction of Ti_3_C_2_ has a negligible effect on the optical coupling output of the device.

In order to explore the effect of Ti_3_C_2_ on the electronic properties of ETL, the energy levels of ZnO/PEI and ZTC/PEI were explored by UPS measurement and the Tauc plots, the corresponding data are given in **Figure** **2**a,b, respectively. It can be seen that the conduction band minimum (CBM) decreased from −3.56  to −3.99 eV  while the bandgap remained unchanged with the increase of Ti_3_C_2_ loading, illustrating that Ti_3_C_2_ played a role of energy level regulation. Here, we designed ITO/ZTC/PEI/CsPb_0.64_Zn_0.36_I_3_/TCTA/MoO_3_/Au device structure (Figure 2c), and the corresponding energy band diagram for all functional layers is shown in Figure 2d. Changes in ZnO energy level can be attributed to the changes in surface termination groups of Ti_3_C_2_ after annealing^[^ ^11^, ^13^ ^]^ (Figure S6, Supporting Information). Typically, electrons are injected into the emitting layer through the CBM of the ETL. The energy level difference between the ETL and EML is an important factor affecting the electron injection efficiency. Previous reports have used PEI as an interface modification layer to improve electron injection efficiency while blocking electron transport. The thickness of PEI as an insulating material must be moderate, otherwise, an excessive thickness will increase device resistance, causing unnecessary thermal effects, thereby reducing the device efficiency. Therefore, balanced charge transfer in the device may not be completely carried out only through adding a PEI layer.^[^ ^14^ ^]^

**Figure 2:**
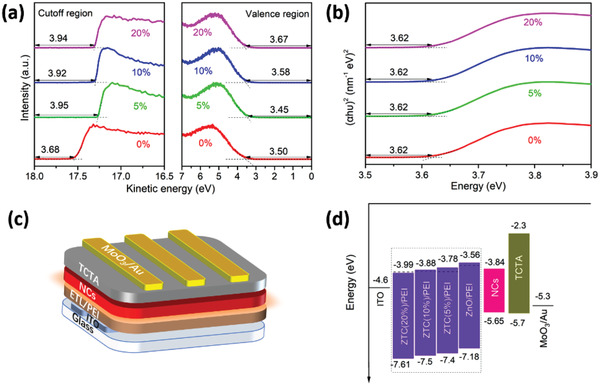
a) UPS spectra, b) Tauc plots of ZnO/PEI film and ZTC/PEI film with different contents of Ti_3_C_2_ (from 5% to 20%) deposited on the ITO glass substrate. c) Schematic of the CsPb_0.64_Zn_0.36_I_3_ perovskite NC LED configuration. d) Device energy‐level diagram for each functional layer in the LEDs.

Through the above discussion of the regulation of ETL energy level by Ti_3_C_2_, we can further envision the effect of which on the carrier transport in the device. The charge transfer processes in perovskite NC LEDs based on different contents of Ti_3_C_2_ in ETL are illustrated in **Figure** **3**. First, for the pristine ZnO‐based device (Figure 3a), there is a large potential well between ETL and EML compared with the one between HTL and EML, which will cause excessive electron accumulation, resulting in carrier recombination imbalance and further affecting device efficiency. At the beginning of the addition of 5% Ti_3_C_2_ (Figure 3b), the potential well between ETL and EML becomes small, but there may still be an accumulation of electrons due to the larger electron difference of ETL. When the Ti_3_C_2_ increases to 10% (Figure 3c), the little potential well between ETL and EML becomes a little potential barrier, which will reduce the electron injection efficiency of ZTC and promote the injection balance of electrons and holes. The accumulation of electrons at the interface between EML and HTL will be suppressed; the space charge will be reduced; the built‐in electric field will be weakened. It may be manifested by an increase in device efficiency.^[^ ^15^ ^]^ When 20% Ti_3_C_2_ is added in ZnO NCs (Figure 3d), the electron injection barrier becomes larger, which will reduce electron injection efficiency and cause charge transport to be once again unbalanced, thus degrading device performance.

**Figure 3:**
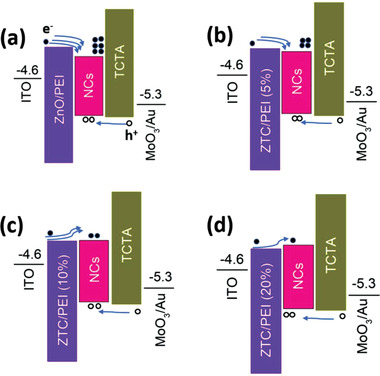
Charge carrier injection and recombination mechanisms for a) 0%, b) 5%, c) 10% and d) 20% Ti_3_C_2_ based devices.

In order to determine the influence of Ti_3_C_2_ on the device performance of perovskite NC LEDs, devices with an architecture of ITO (200 nm) /ETL (70 nm)/CsPb_0.64_Zn_0.36_I_3_ (50 nm)/TCTA (70 nm) /MoO_3_/Au (60 nm)  were fabricated (Figure S7, Supporting Information). The normalized electroluminescence (EL) and PL spectra for all sample devices are shown in Figure S8 (Supporting Information). It is noted that the EL spectra are almost consistent with the PL spectra, illustrating that the EL is indeed from the perovskite NCs without any noticeable contribution from other charge transport materials.^[^ ^16^ ^]^ **Figure** **4**a presents the voltage‐dependent variations of luminance and current density for CsPb_0.64_Zn_0.36_I_3_ NC LEDs with different ETLs. It can be seen that the turn‐on voltages and maximum luminance of these devices show a slight increase as the additional content of Ti_3_C_2_ from 0% to 10%. When the content of Ti_3_C_2_ continuously increased to 20%, the turn‐on voltage became larger and the maximum luminance significantly decreased. The increase in turn‐on voltage is attributed to the fact that electron injection gradually changes from a potential well to a barrier, which means that the electron injection capability is lowered. The increase in luminance is attributed to a more balanced charge carrier recombination at low Ti_3_C_2_ concentrations.^[^ ^6c^ ^]^ When the content of Ti_3_C_2_ increased, the barrier height between ETL and EML further increased, the recombination of charge carriers was again unbalanced, resulting in a sharp drop in luminance. Similar changes were observed in EQE versus current density characters of these four devices (Figure 4b), and the corresponding electrical parameters are summarized in **Table** **1**. These results, in turn, confirm our previous assumptions. The maximum EQE of 17.4% was achieved for a 10% ZTC based device, which is 1.2‐fold higher than 14.2% EQE of pristine ZnO‐based device. Moreover, 10% ZTC‐based device has an efficiency roll‐off of 22.9% at current density of 500 mA  cm^−2^, a 15.8% improvement over 38.7% roll‐off of pristine ZnO‐based device. This may be attributed to the suppression of auger recombination caused by unbalanced charge carrier injection (which favors trion formation at higher current densities).^[^ ^5^, ^15^ ^]^

**Figure 4:**
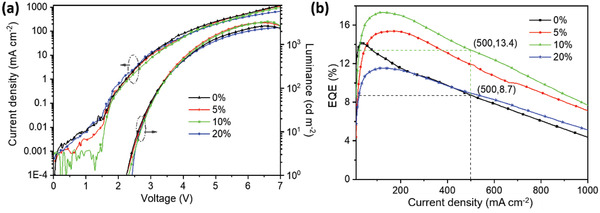
a) Current density and luminance versus bias voltage. b) EQE versus current efficiency of CsPb_0.64_Zn_0.36_I_3_ perovskite NC LEDs with different contents of Ti_3_C_2_.

**Table 1:** Electronic performance of CsPb_0.64_Zn_0.36_I_3_ perovskite NC LEDs with 0–20% Ti_3_C_2_

ETL	EL [nm]	FWHM [nm]	*V*_ON_ [V]	*L*_max_ [cd m^−2^]	EQE_max_ [%]
ZnO	682	33	2.24	2678	14.2
ZTC (5%)	682	33	2.25	3225	15.4
ZTC (10%)	682	33	2.28	3260	17.4
ZTC (20%)	682	33	2.43	2483	11.5

The electron mobility of ZnO films was 2.21 × 10^−3^ cm^2^ V^−1^ s^−1^ which is much higher than that of TCTA (2 × 10^−5^ cm^2^ V^−1^ s^−1^).^[^ ^7^, ^17^ ^]^ The large electron mobility can cause excess electron accumulation at the interface between the EML and the ETL, thereby forming a space charge and causing luminescence quenching, which ultimately leads to a reduction of device efficiency.^[^ ^7b^ ^]^ To further confirm the effect of the addition of Ti_3_C_2_ (electron mobility of 2.23 × 10^−5^ cm^2^ V^−1^ s^−1^)^[^ ^11b^ ^]^ on the carrier transport balance, the electron‐only devices with device structure of ITO/ZTC/PEI (0% and 10%) /perovskite NCs /LiF/Al and the hole‐only devices with device structure of ITO/PEDOT:PSS/perovskite NCs/TCTA/MoO_3_/Au were fabricated. As shown in Figure S9a (Supporting Information), the current density of the electron‐only device with 10% ZTC became lower than that of the electron‐only device without Ti_3_C_2_ and was much closer to that of the hole‐only device, proving that the carrier transport between electron and hole are more balanced. In addition, the electron mobility of different ETL films are estimated by fitting the space‐charge‐limited‐current region (SCLC). As shown in Figure S9b (Supporting Information), the mobility of the added ZnO film with 0% to 20% Ti_3_C_2_ content are 1.98 × 10^−3^ cm^2^ V^−1^ s^−1^, 1.61 × 10^−3^ cm^2^ V^−1^ s^−1^, 1.16 × 10^−3^ cm^2^ V^−1^ s^−1^, 9.03 × 10^−4^ cm^2^ V^−1^ s^−1^, illustrating the addition of Ti_3_C_2_ reduces the electron mobility of ZnO and the current density decreases with the increase of Ti_3_C_2_ addition. As discussed above, we conclude that the effect of Ti_3_C_2_ on carrier transport balance can be divided into two aspects. On the one hand, the addition of Ti_3_C_2_ increases the work function of ETL. On the other hand, the electron mobility of ETL decreases due to the lower electron mobility of Ti_3_C_2_, thus leading to more matched energy levels and carrier mobility between ETL and HTL. Furthermore, the ZTC layer also offers lower surface roughness than that of the ZnO layer. The AFM image of the ZnO film is shown in Figure S10a (Supporting Information), exhibiting root‐mean‐square (RMS) roughness of 3.42 nm.  The RMS roughness is further reduced to 1.49 nm  when 10% of Ti_3_C_2_ was added in the ZnO (Figure S10b, Supporting Information). The uniform ZTC films ensure low leakage current in LEDs and the semilogarithmic curve illustrated in Figure 4a shows that the leakage current of the 10% ZTC‐based device is significantly reduced compared to the original device, which is crucial for LED's performance. Because the reduction of leakage current means the reduction of the current flowing into the device without emission.^[^ ^18^ ^]^ The main works on the red emitting CsPbI_3_ perovskite NC LEDs are summarized in Table S1 (Supporting Information). For now, our devices have achieved the highest EQE. We also tested the repeatability and stability of these devices, as shown in Figure S11 (Supporting Information). The average EQE_max_ of the four types of devices added with 0, 5%, 10%, and 20% Ti_3_C_2_ are 14.2%, 15.3%, 17.3%, and 11.5%, respectively. It can be seen that the LED devices with different Ti_3_C_2_ contents have good repeatability. In addition, the times for LED luminances without Ti_3_C_2_ and with 10% Ti_3_C_2_ drop to half of their initial values are 9 and 23 min,  respectively, illustrating a better charge transport balance can reduce the damage to the device.

In conclusion, conductive sheet‐like Ti_3_C_2_ Mxene was prepared and mixture of Ti_3_C_2_ and ZnO NCs were first utilized as the ETL for perovskite NCs LEDs. On the one hand, the addition of Ti_3_C_2_ achieves charge carrier balance between ETL and HTL by changing the work function and electron mobility of ZnO. On the other hand, the ZTC film also offers lower surface roughness, thus guaranteeing uniform distribution of the perovskite NCs layer for leakage current reduction. As a result, a relatively high EQE of 17.4% and lower efficiency roll‐off were achieved by using ZTC (10%) as the ETL, which is 22.5% improvement compared with the pristine ZnO‐based device (14.2% EQE), showing a great promise for the applications in future display and lighting.

## Conflict of Interest

The authors declare no conflict of interest.

## Supporting information

Supporting Information
