# Supplementary material for: ZnO–Ti3C2 MXene Electron Transport Layer for High External Quantum Efficiency Perovskite Nanocrystal Light‐Emitting Diodes
Source: Adv Sci (Weinh). 2020 Aug 16;7(19):2001562. doi: 10.1002/advs.202001562 (PMC7539190; doi:10.1002/advs.202001562)
Supplement: Supplementary file 1 — Supporting Information [file ADVS-7-2001562-s001.pdf]

© 2020 Wiley-VCH GmbH

ADVANCED SCIENCE
Open Access

Supporting Information

for *Adv. Sci.,* DOI: 10.1002/advs.202001562

ZnO–Ti_3C_2 MXene Electron Transport Layer for High External Quantum Efficiency Perovskite Nanocrystal Light-Emitting Diodes

Po Lu, Jinlei Wu, Xinyu Shen, Xupeng Gao, Zhifeng Shi, Min Lu, William W. Yu,* and Yu Zhang**

$ZnO–Ti_3C_2$ MXene electron transport layer for high external quantum efficiency perovskite nanocrystal light-emitting diodes

Po Lu[1], *Jinlei Wu*[1], *Xinyu Shen*[1], *Xupeng Gao, Zhifeng Shi, Min Lu*, William W. Yu*, Yu Zhang**

P. Lu, J. Wu, X. Shen, X. Gao, M. Lu, Prof. W. W. Yu, Prof. Y. Zhang

State Key Laboratory of Integrated Optoelectronics and College of Electronic Science and Engineering Jilin University Changchun 130012, China.

Prof. Z. Shi

Key Laboratory of Materials Physics of Ministry of Education, School of Physics and Microelectronics, Zhengzhou University, Zhengzhou 450052, China.

Prof. W. W. Yu

Department of Chemistry and Physics Louisiana State University Shreveport, LA 71115, USA.

*E-mails: lumin_1992@163.com (M.L.); wyu6000@gmail.com (W.W.Y.); yuzhang@jlu.edu.cn (Y.Z.).

Experimental section

Materials

Cs_2CO_3 (99.9%) was bought from J&K Chemicals. Oleic acid (OA, 90%), octadecene (ODE, 90%) and lead (II) iodide (PbI_2, 99.999%) were obtained from Sigma-Aldrich. Zinc (II) iodide (ZnI_2, 99.99%), oleylamine (OLA, 70%), zinc acetate ($Zn(Ac)_2$, 99.999%) and sodium hydroxide were purchased from Aladdin. Toluene (99.5%), ethanol (99%), acetone (95%), hydrochloric acid and lithium fluoride (LiF) were obtained from Beijing Chemical Factory. Ti_3AlC_2 (≥ 98 wt %) powders were purchased from Jilin 11th Technology Co., Ltd. All chemicals were used directly without further purification.

Preparation of Ti_3C_2 MXene

Firstly, 1 g of LiF was added to 20 mL of 9 M HCl and stirred for 5 min, then 1 g of Ti_3AlC_2 was slowly added into the mixed solution over another 5 minutes to avoid a violent reaction. The solution was maintained for 24 h at 35 °C by continuous stirring. The reaction product was separated by centrifugation at 3000 rpm for 10 min and this was repeated 5 times until the pH value reached 6. After that, the precipitate was dispersed into 100 mL deionized water. This suspension was purged with N_2 for 30 min to drive away the residual air, sealed and ultrasonicated for 5 h in an ice bath. Finally, the solution was centrifuged for 10 min at 8000 rpm to get a colloidal solution of delaminated $Ti_3C_2T_x$ MXene nanosheets. The concentration was estimated to be ~0.5 mg/mL.

Synthesis of ZnO NCs

ZnO NCs were synthesized based on a previous report.[1] Firstly, 0.4403 g zinc acetate and 30 mL ethyl alcohol were added into a 100 ml three-neck flask, degassed for 10 min at room temperature. Then the solution was heated to 80 °C for 30 min until zinc acetate

powder was completely dissolved. After that, the flask was cooled to room temperature naturally. A base solution (0.2 g sodium hydroxide dissolved in 10 mL ethyl alcohol) was quickly injected into the reaction solution, and the mixture was kept stirring for 4 h at room temperature under N_2. After centrifuging, the obtained products were dissolved in ethyl alcohol.

Preparation of ZnO–Ti_3C_2 MXene composites

200 μL $Ti_3C_2T_x$ MXene colloidal solution was added into 10 mL ethyl alcohol and then ultrasonicated for 30 min in an ice bath. Then the $Ti_3C_2T_x$ MXene solution was directly mixed with ZnO NCs (50 mg/mL) according to the volume ratios (0, 0.05, 0.1, 0.20), and was stirred for 5 min under N_2. The mixed solution was used as ETL.

Synthesis of $CsPb_{0.64}Zn_{0.36}I_3$ perovskite NCs

The synthesis of $CsPb_{0.64}Zn_{0.36}I_3$ perovskite NCs based on previous work.[2] PbI_2 (0.173 g), ZnI_2 (0.12 g), ODE (10 mL), OLA (2 mL) and OA (2 mL) were loaded into a 50 mL three-neck flask and heated to 120 °C under vacuum for 1 h. Then the temperature was increased to 170 °C and 1.5 mL Cs-oleate solution (cesium oleate was prepared by adding Cs_2CO_3 (0.814 g), OA (2.5 mL), and ODE (30.0 mL) into a 100 mL three-neck flask; it was degassed and dried under vacuum for 1 h at 120 °C, and heated to 150 °C under N_2 until a clear solution was obtained) was quickly injected into the three-neck flask. Five seconds later, the solution was immediately cooled down to room temperature by an ice-water bath. The $CsPb_{0.64}Zn_{0.36}I_3$ NCs were centrifuged at 5 000 rpm for 10 min; the precipitate was reserved and dissolved in equal volumes of toluene and ethyl acetate to be further purified by centrifuging at 10 000 rpm for 5 min. Eventually, the NCs were dispersed in toluene for use.

Device fabrication

Indium tin oxide (ITO) glass substrates were cleaned by UV-ozone treatment for 10 min. The ETL was prepared via spin-coating ZnO NC or ZnO–Ti_3C_2 composite solution onto the ITO substrates at 1000 rpm for 40 s and annealed in air at 150 °C for 10 min. Then, a solution of polyethyleneimine (PEI) (dissolved in 2-methoxyethanol, 0.2% mass ratio) was spin-coated onto the ZnO or ZnO–Ti_3C_2 film at a speed of 3000 rpm for 50 s and annealed at 125 °C for 10 min in the glovebox. Perovskite NC emitting layer (20 mg mL^{-1}) was then deposited by spin-coating at 2000 rpm for 50 s. 4,4',4"-tris(carbazol-9-yl)-triphenylamine (TCTA), MoO_3 and Au were then sequentially deposited by thermal evaporation in a vacuum deposition chamber (1×10^{-7} Torr).

Characterizations

Absorption spectra were measured using a Shimadzu UV-2550 spectrophotometer. The PL spectra of the perovskite NCs and the EL spectra of LEDs were measured using an S3 Ocean Optics spectrometer. The morphology of the perovskite NCs, Ti_3C_2 Mexene were observed with a JEM-2100F transmission electron microscope (TEM). Atomic force microscopy (AFM) images were recorded using a VEECO DICP-II microscope. X-ray diffraction (XRD) patterns were acquired using a Bruker D8 Advance X diffractometer (Cu Kα, λ = 1.5406 Å). The energy levels of ZnO and ZnO–Ti_3C_2 were measured using an integrated ultrahigh vacuum system equipped with a multi-technique surface analysis system (VG Scienta R3000) with excitation energy of 21.218 eV and were determined by ultraviolet photoelectron spectroscopy (UPS). The current-voltage characteristics of the devices were measured with a Keithley 2612B source meter and the LED brightness was determined using a Photo Research Spectra Scan spectrometer PR650.

Energy level calculation of ETL films

The work function (WF) is calculated by a formula as

WF = hν - E

where hν is photo energy (21.2 eV), E is calculated from the high-binding energy secondary electron cutoff region shown in Figure 2a. The energy of valance band (E_{VB}) is calculated by a formula as

$E_{VB} = E_F - E_1$

Where E_F is the fermi lever, equals –WF, and E_1 is calculated from the valance band edge region shown in Figure 2a. The energy of values of conduction band minimum (E_{CB}) were determined by a formula as

$E_{CB} = E_{VB} + E_g$

Where Eg is band gap of ZTC/PEI films.

Calculation of electron mobility

The electron mobility of diffreent ETL films are estimated by fitting the space-charge-limited-current region (SCLC) with Mott–Gurney law $J = 9\varepsilon_0\varepsilon_r\mu V^2/8L^3$, where ε_0 is the vacuum permittivity; ε_r is the average relative dielectric constant of ZnO ($\varepsilon_r \approx 4$); L is the thickness of the ETL film; and J, μ, and V are the measured current density, carrier mobility, and applied voltage, respectively.

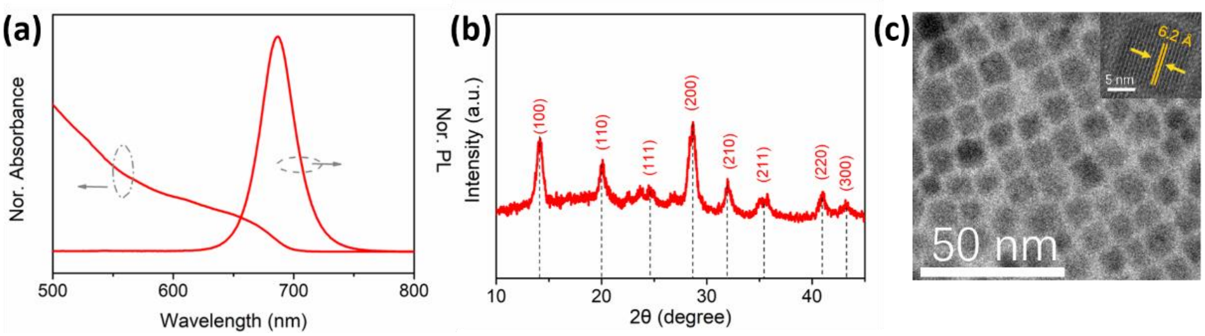

Figure S1. (a) Absorption and PL spectra, (b) XRD pattern and (c) TEM image of $CsPb_{0.64}Zn_{0.36}I_3$ perovskite NCs. Inset is an HRTEM image.

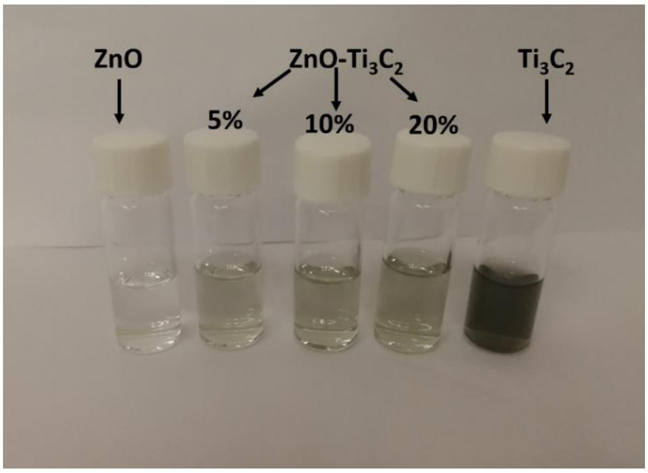

Figure S2. Digital photo of solutions of ZnO NCs, Ti_3C_2, and their mixtures.

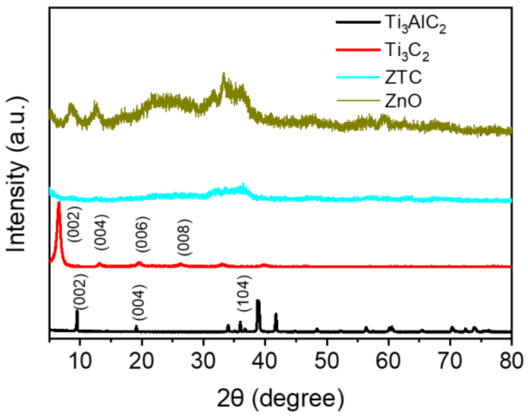

Figure S3. XRD patterns of Ti_3AlC_2, Ti_3C_2, ZTC and ZnO.

Note. It can be seen that the XRD pattern of ZnO is consistent with our previous work.[1] However, due to the small amount of Ti_3C_2, the peaks of Ti_3C_2 are hardly visible in the XRD pattern of ZTC.

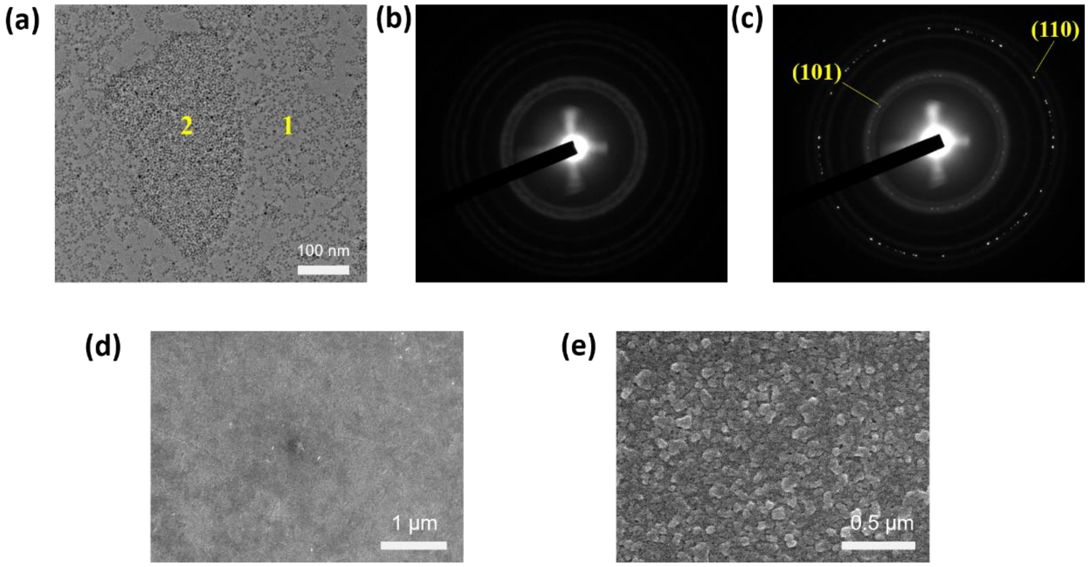

Figure S4. (a) TEM image of mixture of ZnO and Ti_3C_2. (b) Diffraction pattern of area 1 in (a). (c) Diffraction pattern of area 2 in (a). Top-view SEM images of unannealed (d) and annealed (e) mixture of Ti_3C_2 and ZnO films.

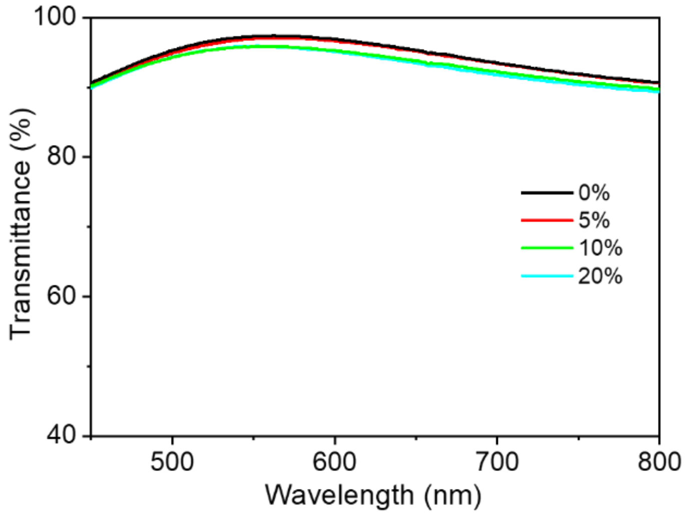

Figure S5. Transmittance of ZTC films with different contents of Ti_3C_2.

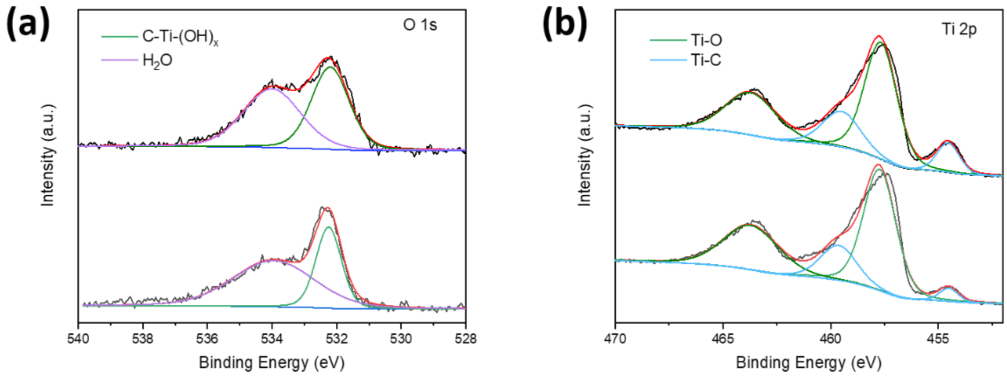

Figure S6. (a) O 1s, (b) Ti 2p core level XPS spectra of unannealed (bottom) and annealed (top) Ti_3C_2, respectively.

Note. It can be seen that the surface termination groups increase after annealing. Due to the small amount of Ti_3C_2 added, the fine structure cannot be detected. We can assume that also the Ti_3C_2 dispersed in the ZnO matrix undergoes a similar process when annealed in air and the amount of terminating group changes with different Ti_3C_2 addition.

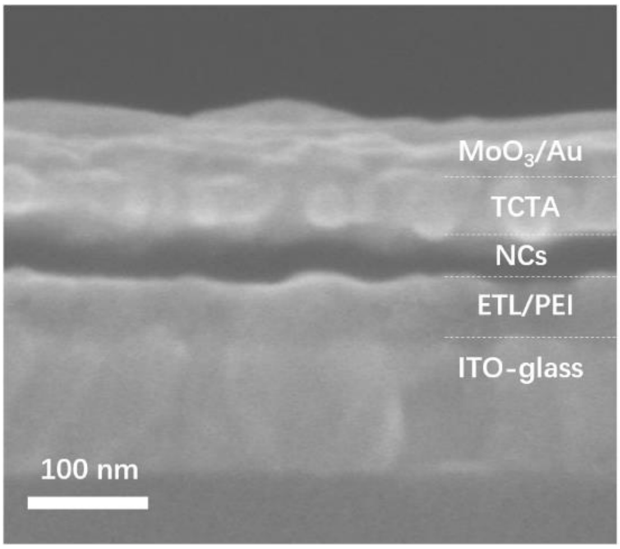

Figure S7. Cross-section SEM image of the device structure.

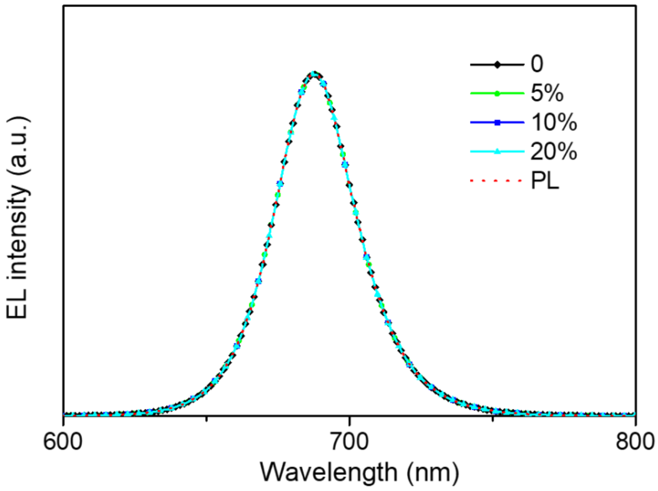

Figure S8. PL for $CsPb_{0.64}Zn_{0.36}I_3$ perovskite NC films and EL spectra for $CsPb_{0.64}Zn_{0.36}I_3$ perovskite NC LEDs with different contents of Ti_3C_2.

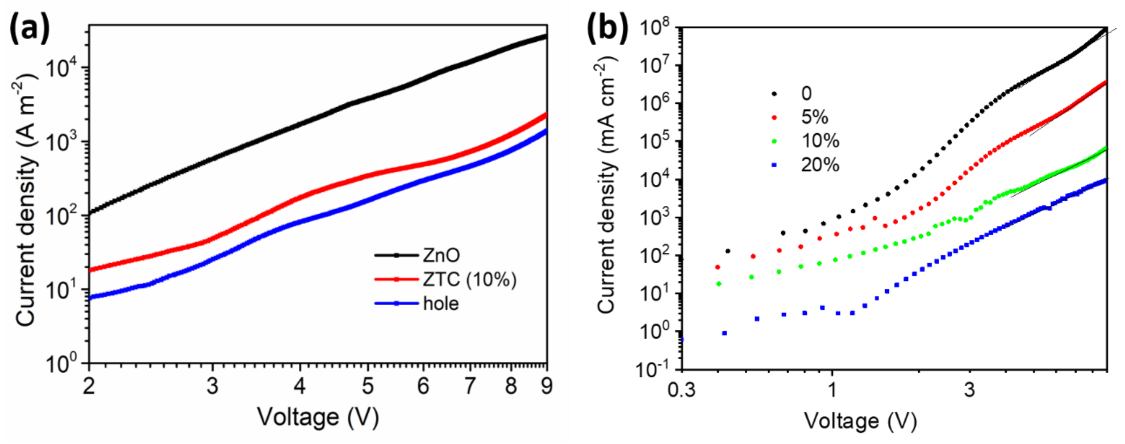

Figure S9. (a) Current densities as a function of bias voltage of electron-only (black) and hole-only (blue) devices. The electron-only device structure is ITO / ZnO / PEI /perovskite NCs /LiF/Al and ITO / ZTC (10%) / PEI / perovskite NCs / LiF/Al (red); the hole-only device structure is ITO / PEDOT:PSS / perovskite NCs / TCTA / MoO_3 / Au. (b) Current densities as a function of bias voltage of electron-only devices. The electron-only device structure is ITO/ZTC (0% - 20%)/PEI /LiF/Al.

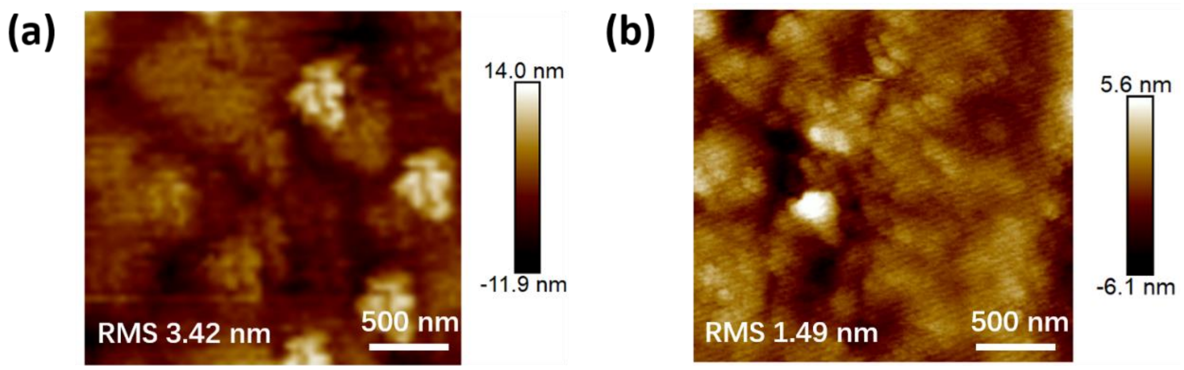

Figure S10. AFM images of (a) ZnO/PEI and (b) ZTC (10%) / PEI films.

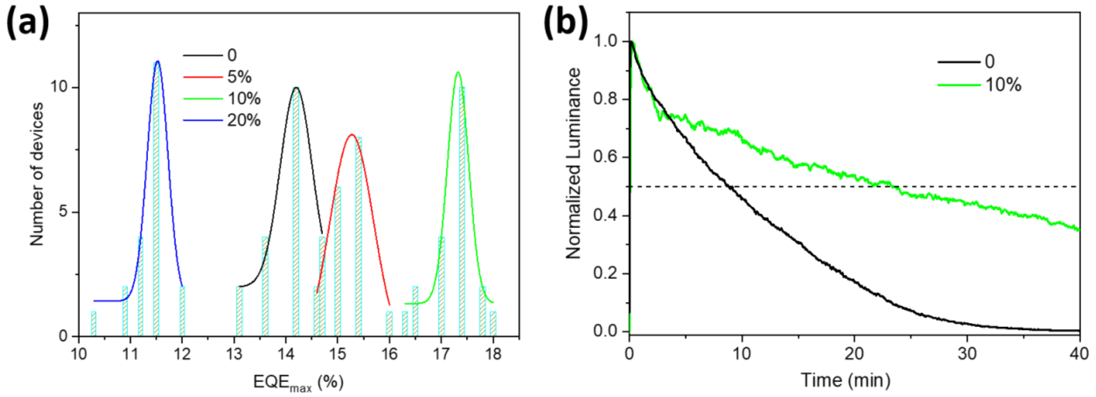

Figure S11. (a) EQE reproducibility of LED devices with different amounts of Ti_3C_2 added. (b) Stability tests for LEDs with different amounts of Ti_3C_2 added under 3 V drving voltage.

Table S1. Performance of LEDs based on all-inorganic perovskite NCs

Year	Material	EL (nm)	EQE_{max} (%)	CE (cd A^{-1})	L_{max} (cd m^{-2})	V_{on} (V)	Ref.
2016	$CsPbI_3$	698	5.7		206	2.1	[3]
2016	$CsPbI_3$	688	7.25	0.49	435	1.9	[4]
2017	$CsPbI_3$	695	0.04		27		[5]
2017	$CsPbI_3$	680	0.21	0.012	7.2	3	[6]
2017	$CsPbI_3$	683	7.3		100	3.3	[7]
2017	$CsPbI_3$	688	5.02		748	4.1	[8]
2018	$CsPbI_3$	660	0.25	0.84	217	3	[9]
2018	$CsPbI_3$	690	11.2		11.6	2.2	[10]
2018	$CsPbI_3$	690	14.08		1444	3.5	[11]
2018	$CsPbI_3$	692	8.2	0.8	827	2.0	[12]
2018	$CsPbI_3$	691	13.5		1152	2	[13]
2019	$CsPb_{0.64}Zn_{0.36}I_3$	682	15.1		2202	2.0	[2]
2019	$CsPbI_3$	691	10.3		823	2.2	[14]
2020	$CsPbI_3$	689	10.6		981	2.1	[15]
2020	$CsPbI_3$	686	13.7		14725	2.1	[16]
2020	$CsPbI_3$	689	1.25		468	4.0	[17]
2020	$CsPbI_3$	680	2.67		178		[18]
2020	$CsPbI_3$	684	10.3		1605	2.3	[19]
2020	$CsPbI_3$	685	12.7		6000	1.95	[20]
2020	$CsPb_{1-x}Zn_xI_3$	687	13.7		3714	2.20	[21]
	$CsPbI_3$	690	6.9		1112	2.20	
2020	**$CsPb_{0.64}Zn_{0.36}I_3$**	**682**	**17.4**		**3531**	**2.28**	**This work**

Abbreviations: CE, current efficiency; EL, electroluminescence wavelength.

References

[1] H. Wu, Y. Zhang, X. Zhang, M. Lu, C. Sun, T. Zhang, W. W. Yu, *Adv. Opt. Mater.* **2017**, 5, 1700377.

[2] X. Shen, Y. Zhang, S. V. Kershaw, T. Li, C. Wang, X. Zhang, W. Wang, D. Li, Y. Wang, M. Lu, L. Zhang, C. Sun, D. Zhao, G. Qin, X. Bai, W. W. Yu, A. L. Rogach, *Nano Lett.* **2019**, 19, 1552.

[3] G. Li, F. W. R. Rivarola, N. J. L. K. Davis, S. Bai, T. C. Jellicoe, F. de la Peña, S. Hou, C. Ducati, F. Gao, R. H. Friend, N. C. Greenham, Z.-K. Tan, *Adv. Mater.* **2016**, 28, 3528.

[4] X. Zhang, C. Sun, Y. Zhang, H. Wu, C. Ji, Y. Chuai, P. Wang, S. Wen, C. Zhang, W. W. Yu, *The Journal of Physical Chemistry Letters* **2016**, 7, 4602.

[5] N. J. L. K. Davis, F. J. de la Peña, M. Tabachnyk, J. M. Richter, R. D. Lamboll, E. P. Booker, F. Wisnivesky Rocca Rivarola, J. T. Griffiths, C. Ducati, S. M. Menke, F. Deschler, N. C. Greenham, *The Journal of Physical Chemistry C* **2017**, 121, 3790.

[6] C. Zou, C.-Y. Huang, E. M. Sanehira, J. M. Luther, L. Y. Lin, *Nanotechnology* **2017**, 28, 455201.

[7] J. Si, Y. Liu, Z. He, H. Du, K. Du, D. Chen, J. Li, M. Xu, H. Tian, H. He, D. Di, C. Lin, Y. Cheng, J. Wang, Y. Jin, *ACS Nano* **2017**, 11, 11100.

[8] J. Pan, Y. Shang, J. Yin, M. De Bastiani, W. Peng, I. Dursun, L. Sinatra, A. M. El-Zohry, M. N. Hedhili, A.-H. Emwas, O. F. Mohammed, Z. Ning, O. M. Bakr, *J. Am. Chem. Soc.* **2018**, 140, 562.

[9] Y.-H. Suh, T. Kim, J. W. Choi, C.-L. Lee, J. Park, *ACS Appl. Nano Mater.* **2018**, 1, 488.

[10] M. Lu, X. Zhang, X. Bai, H. Wu, X. Shen, Y. Zhang, W. Zhang, W. Zheng, H. Song, W. W. Yu, A. L. Rogach, *ACS Energy Lett.* **2018**, 3, 1571.

[11] G. Li, J. Huang, H. Zhu, Y. Li, J.-X. Tang, Y. Jiang, *Chem. Mater.* **2018**, 30, 6099.

[12] M. Lu, H. Wu, X. Zhang, H. Wang, Y. Hu, V. L. Colvin, Y. Zhang, W. W. Yu, *ChemNanoMat* **2019**, 5, 313.

[13] M. Lu, X. Zhang, Y. Zhang, J. Guo, X. Shen, W. W. Yu, A. L. Rogach, *Adv. Mater.* **2018**, 30, 1804691.

[14] M. Lu, J. Guo, P. Lu, L. Zhang, Y. Zhang, Q. Dai, Y. Hu, V. L. Colvin, W. W. Yu, *J. Phys. Chem. C* **2019**, 123, 22787.

[15] Y. Zhu, J. Zhao, G. Yang, X. Xu, G. Pan, *Nanoscale* **2020**, 12, 7712.

[16] M. Lu, J. Guo, S. Sun, P. Lu, J. Wu, Y. Wang, S. V. Kershaw, W. W. Yu, A. L. Rogach, Y. Zhang, *Nano Lett.* **2020**, 20, 2829.

[17] Y. Huang, W. Luan, M. Liu, L. Turyanska, *J. Mater. Chem. C* **2020**, 8, 2381.

[18] Y. Wei, X. Li, Y. Chen, Z. Cheng, H. Xiao, X. Li, J. Ding, J. Lin, *J. Phys. Chem. Lett.* **2020**, 11, 1154.

[19] X. Zhang, Q. Zeng, Y. Xiong, T. Ji, C. Wang, X. Shen, M. Lu, H. Wang, S. Wen, Y. Zhang, X. Yang, X. Ge, W. Zhang, A. P. Litvin, A. V. Baranov, D. Yao, H. Zhang, B. Yang, A. L. Rogach, W. Zheng, *Adv. Funct. Mater.* **2020**, 30, 1910530.

[20] Y. Wang, Y. Teng, P. Lu, X. Shen, P. Jia, M. Lu, Z. Shi, B. Dong, W. W. Yu, Y. Zhang, *Adv. Funct. Mater.* **2020**, 30, 1910140.

[21] X. Shen, X. Zhang, C. Tang, X. Zhang, P. Lu, Z. Shi, W. Xie, W. W. Yu, Y. Zhang, *J. Phys. Chem. Lett.* **2020**, 11, 3853.
